# 

**Published:** 1882-02

**Authors:** 


					﻿NOTICE.
All articles fOv publication, books for t*eifiew, business communications, etc.,
must be sent to Editor, 2109 Chestnut tytreet, Philadelphia.
Subscribers who have not paid their Subscrip-
tions will please giye the' matter .their-?
. prompt attention. .
Authors of accepted papers are furnished with copies of the
number in which their articles appear; application for such copies
to be made when sending papers. Any irregularity in the receipt
of this Journal should be promptly reported to,Editor.
WANTED-^-Copies of the January (1881) numbei•. Thirty cents
paid at this office, for sound copies.
				

